# Temporoparietal brain structures support sentence processing across the adult lifespan

**DOI:** 10.1162/IMAG.a.1009

**Published:** 2025-11-13

**Authors:** Nicholas Riccardi, Alex Teghipco, Ida Rangus, Saanvi Somani, Sarah Newman-Norlund, Roger Newman-Norlund, Chris Rorden, Julius Fridriksson, Leonardo Bonilha, William Matchin

**Affiliations:** Department of Communication Sciences and Disorders, University of South Carolina, Columbia, SC, United States; Center for Stroke Research Berlin, Berlin, Germany; Department of Psychology, University of South Carolina, Columbia, SC, United States; Department of Neurology, School of Medicine Columbia, University of South Carolina, Columbia, SC, United States

**Keywords:** aging, MRI, language, syntax, mediation analysis

## Abstract

Although older adults maintain most language abilities, they can experience declines in complex sentence comprehension. How brain structure and function mediate age-related changes in sentence processing remains underspecified. Here, using a novel neuroimaging approach to perform region-based mediation statistics, we investigated the neural correlates of memory capacity and sentence processing in 187 neurologically intact adults aged 20–80 years. Working and auditory–verbal short-term memory were measured using two National Institutes of Health Cognitive Toolbox tasks, and reading sentence processing was measured with a self-paced reading (SPR) task consisting of closely matched noncanonical (object-relative; OR) and canonical (subject-relative; SR) conditions. Behaviorally, we found that memory capacity mediated the relationship between age and overall sentence comprehension. Age, and not memory capacity, was the most important factor for sentence processing speed. Neuroanatomically, we found that bilateral gray matter volume (GMV; structure) in posterior temporal/inferior parietal areas and partial amplitude of low-frequency fluctuations (pALFF; function) in the left SMG mediated age-related processing speed for noncanonical compared with canonical conditions after controlling for memory capacity. These findings suggest that bilateral temporoparietal brain areas support the processing of complex syntactic structures across the lifespan in ways that are partially independent of memory abilities.

## 1 Introduction

Language is a cornerstone of human interaction and well-being. While general language abilities are usually well preserved in neurologically intact aging (Horton et al., 2010), older adults can experience declines in sentence processing (Burke et al., 2000; Peelle, 2019). This can discourage them from engaging in reading or conversation, resulting in reduced quality of life or accelerated cognitive decline (Burke & Shafto, 2011). The goal of this retrospective analysis was to investigate neurobiological substrates of memory capacity and sentence processing decline across the adult lifespan in a cohort of 187 neurologically intact individuals aged 20–80 years.

Sentence processing relies on multiple cognitive functions working in parallel, including lexical-semantics, hierarchical structure building, and working memory (Desai & Riccardi, 2021; Kemmerer, 2022). A large body of research has been devoted to understanding how working memory, and especially verbal working memory, is related to sentence comprehension (Caplan & Waters, 1999, 2013; Carpenter & Just, 2013; DeDe et al., 2004; Just & Carpenter, 1992; Just et al., 1996; King & Just, 1991; Miyake et al., 1994; Van der Linden et al., 1999; Waters & Caplan, 1996a, 2001, 2004; Waters et al., 2016). Certain linguistic structures, such as object-relative sentences with noncanonical word orders (e.g., *the boy that the girl chased is happy*), are known to increase processing difficulty (Chomsky & Miller, 1968; Fedorenko et al., 2007; Miller & Chomsky, 1963; Vasishth & Engelmann, 2021). Older adults often experience declines in working memory capacity, which can impact their ability to process complex sentences efficiently (Grossman et al., 2002; Lewis et al., 2006; Salis, 2011; Small et al., 2000; Stowe et al., 1998). Prior work has shown that older adults tend to be slower or less accurate than younger adults when processing sentences in general (Davis & Ball, 1989; Norman et al., 1992; Stine-Morrow et al., 1996; Wingfield et al., 2003), with difficulties more pronounced for syntactically complex constructions (Liu & Wang, 2019; Meyer, 1987; Obler et al., 1991; Poulisse et al., 2019; Zhu et al., 2018).

Studies have used both online and offline metrics to measure sentence processing (Caplan & Waters, 1999, 2013; DeDe et al., 2004; Gilardone et al., 2024; Marvel & Desmond, 2012; Van der Linden et al., 1999). Online measures, such as reaction times during reading, provide insights into real-time cognitive processes involved in language comprehension. Offline measures, such as comprehension questions after a sentence, assess end products of sentence processing which could reflect both online operations during sentence reading or “after the fact” aspects such as memory retrieval. The specific relationships between online/offline sentence processing, memory capacity, and aging remain unclear. Some theories propose that declines in general working memory resources account for age-related differences in syntactic processing, as reduced memory capacity limits the ability of older adults to maintain and manipulate linguistic information (Carpenter & Just, 2013; Just & Carpenter, 1992; Just et al., 1996; Miyake et al., 1994). Conversely, other work suggests that there may be a separate system for syntactic parsing that is at least partially dissociable from domain-general working memory abilities (Caplan & Waters, 1999, 2013; Waters & Caplan, 1996a; Waters et al., 2016; ). For example, while age-related deficits in sentence processing are present in both off- and online metrics, online declines may be attributable to language-specific operations whereas offline performance is related to domain-general resources such as working memory (Caplan & Waters, 1999; DeDe et al., 2004; Van der Linden et al., 1999; Waters & Caplan, 2004). In this context, neuroimaging offers a powerful approach to explore shared or dissociable neural substrates related to memory capacity and sentence processing across the lifespan.

Neuroimaging and neuropsychological studies have revealed a distributed, largely left-lateralized frontal and temporoparietal network that supports sentence processing and related operations (Friederici, 2011; Hagoort & Indefrey, 2014; Matchin & Hickok, 2020; Meyer & Friederici, 2016). However, investigations examining age-related changes to these networks have suggested that language and cognition may increasingly rely on the right hemisphere as individuals age (Baciu et al., 2016; Kang et al., 2021; Lee et al., 2022; Peelle, 2019), although the findings are somewhat incongruous. For example, Tyler et al. (2010) found that despite age-related atrophy, preserved syntactic processing in older adults was associated with upregulation of right hemisphere frontotemporal regions, suggesting a supporting/compensatory role for the right hemisphere. Conversely, Antonenko et al. (2013) showed that functional connectivity to right hemisphere language homologues was associated with worse syntactic processing in older adults, suggesting that right hemisphere upregulation may be maladaptive. Finally, Campbell et al. (2016) found no evidence of functional reorganization in older adults during syntactic processing and observed that syntactic processing in older adults was relatively unaffected by widespread age-related declines in gray matter. These contradictory findings highlight an ongoing debate about the role of brain structure and function in syntactic processing across the lifespan, especially regarding (1) whether age-related changes in brain structure are related to sentence processing declines and (2) functional compensation versus maladaptation in left frontotemporal language regions or right hemisphere language homologues.

It also remains unclear which brain areas in older adults support sentence processing through their roles in more domain-general processes, such as working memory or auditory–verbal STM, versus more language-specific functions. For example, regions such as the inferior frontal cortex and supramarginal gyrus may contribute through their roles in working memory or auditory–verbal STM (Guidali et al., 2019; Pisoni et al., 2019; Rogalsky & Hickok, 2011; Rogalsky et al., 2008), while posterior middle and superior temporal gyri have been postulated as being language specific (Matchin, 2018; Matchin & Hickok, 2020; Matchin et al., 2022). This can be tested by examining whether brain structure or function in these areas is shared or partially dissociable neural substrates for memory capacity and sentence processing.

Here, we examined behavioral and neuroanatomical relationships between age, memory capacity (using separate working memory and auditory–verbal STM tasks), and sentence processing measured with an adapted self-paced reading (SPR (Fedorenko et al., 2007)) task. We distinguish working memory, which involves the active maintenance and manipulation of information, from auditory–verbal STM, which emphasizes temporary storage in the phonological loop (Caplan & Waters, 1999, 2013; Carpenter & Just, 2013; DeDe et al., 2004; Just et al., 1996; King & Just, 1991; Van der Linden et al., 1999; Waters & Caplan, 2001). The SPR included two matched syntactic conditions: canonical (subject-relative—SR; *The journalist who complimented the editor revised the article for the newspaper*) and noncanonical (object-relative—OR; *The journalist who the editor complimented revised the article for the newspaper*) structures. This design allowed us to investigate overall sentence processing and directly compare the two conditions to assess syntactic complexity. We had three specific aims.

First, although previous studies have described correlational relationships between age, memory capacity, and sentence processing, it is less established whether memory abilities explain age-related declines in sentence processing. Using mediation analysis applied to behavioral data, we tested the extent to which working memory or verbal STM mediates the relationship between aging and sentence processing, measured via offline (comprehension accuracy) and online (response times at the relative clause) metrics. Based on the previously discussed literature, we hypothesized that memory capacity would mediate the relationship between age and offline, but not online, sentence metrics, indicating that reduced memory capacity especially contributes to age-related difficulties in comprehension.

Second, we used a novel and open-source toolbox developed by our group to conduct brain-based mediation analyses (graphical Brain Association Tool; gBAT (Rangus et al., 2024)). Specifically, we examined how brain structure (gray matter volume; GMV) and function (partial amplitude of low-frequency fluctuations; pALFF (Zou et al., 2008)) mediate the relationship between age and overall sentence processing abilities, as measured via online and offline metrics. pALFF measures the regional intensity of spontaneous fluctuations in blood-oxygen-level-dependent (BOLD) signals. When collected in the absence of a task^1^, as in the current study, it is interpreted as a measure of intrinsic brain activity. We hypothesized that reduced GMV in left hemisphere frontotemporal language regions would mediate between age and overall sentence processing performance in offline and online metrics, and that increased pALFF in right hemisphere language homologues would be related to better performance in older adults, indicating possible compensatory upregulation.

Third, we tested whether GMV or pALFF mediated the relationship between age and processing specifically of syntactically complex sentences (OR compared with SR conditions), and examined whether memory capacity was associated with overlapping neural substrates. We hypothesized that left frontotemporal GMV would be related to both offline complex syntactic comprehension and memory capacity, suggesting a shared neural substrate between comprehension and memory. In contrast, we predicted that left posterior temporal GMV would be specifically related to online sentence processing, supporting a role for these areas in real-time hierarchical structure building that is partially independent from memory capacity. For pALFF, we predicted that increased activity in right hemisphere frontotemporal cortices and posterior temporal gyri would be related to better memory capacity and sentence processing, respectively.

## 2 Materials and Methods

### 2.1 Participants

Participants (N=187) aged 20–80 years (mean = 48.5 yo; Table 1) were recruited as part of the ongoing Aging Brain Cohort at the University of South Carolina (ABC@USC (Newman-Norlund et al., 2021)) cross-sectional project and completed the SPR. The primary aim of ABC@USC is to examine cardiovascular, genetic, and lifestyle determinants of brain health across the adult lifespan, with a recruitment goal of 800 participants. As such, the research presented here represents a retrospective sub-analysis, as opposed to a primary research aim of the larger project. All study procedures were approved by the institutional review board of the University of South Carolina. Written informed consent was obtained from each participant. All ethical regulations relevant to human research participants were followed.

**Table 1.** Demographic information and behavioral scores.

	Mean or % (standard deviation)
Age, years	48.5 (19.9)
Sex, female	74%
Level of education (1 = middle school, 6 = graduate degree)	5.1 (1)
Behavioral	
Object-relative accuracy	86.6% (8.4%)
Subject-relative accuracy	89.7% (8.8%)
Object-relative relative clause RT (s)	2.89 (1.28)
Subject-relative relative clause RT (s)	2.43 (.99)
NIH-WM	107.96 (13.88)
NIH auditory–verbal	26.17 (6.39)

Note: Normative data (Slotkin et al., 2012; Weintraub et al., 2013) for NIH-WM and Auditory Verbal Tests show that mean NIH-WM scores for 18–29 years is 111.5 and declines to 94.8 by ages 70–85 years, while NIH Auditory Verbal has a mean score of 27.4 declining to 20.3 by the age of 80 years.

Neuroimaging and behavioral data were collected in different sessions, with the majority of participants completing both sessions within the same week (mean = 5.6 days). ABC@USC focuses on “healthy” or neurologically intact aging and has the following exclusion criteria: history of stroke, neurodegenerative disease, serious acute or chronic conditions that would limit their ability to participate, any severe current illnesses (e.g., cancer), any psychiatric or cognitive diagnoses (e.g., schizophrenia, mild cognitive impairment), or anyone with a BMI of >42 kg/m^2^. All participants had normal or corrected-to-normal vision, and we verbally confirmed that they could hear and comprehend all instructions. All participants who were native English speakers were included in the behavioral analysis. Participants completed the Montreal Cognitive Assessment as part of the test battery (mean score = 27.54; SD = 2.2), confirming that the sample was cognitively intact based on the recommendation of a score of 23 being at-risk for MCI (Carson et al., 2018). Six participants were excluded due to having SPR response times or accuracies > 3 standard deviations from the mean in both SR and OR conditions (final behavioral N = 181). Of these, 169 participants had available structural scans and 155 had available functional scans.

### 2.2 Procedure

#### 2.2.1 Self-paced reading task

Outside of the scanner, participants completed a modified version of the self-paced reading task employed by Fedorenko et al. (2007), from which sentence materials were taken. SPR follows the tradition of using self-paced paradigms to assess language processing (Daneman & Carpenter, 1980; Gygax et al., 2007; Just et al., 1982; Waters & Caplan, 1996b). Two different sentence structures were used: subject-relative (SR; e.g., *The journalist who complimented the editor revised the article for the newspaper*) and object-relative (OR; e.g., *The journalist who the editor complimented revised the article for the newspaper*). There were 24 trials per sentence structure for a total of 48 trials throughout the experiment, presented in random order for each participant. The set of sentences for the SR and OR conditions was identical except for the order of words in the relative clause; each participant was presented with both the SR and OR versions of each sentence. Each sentence was presented visually in the center of the screen, region-by-region such that all words of a given region were presented at the same time, in normal reading order: subject (e.g. *the journalist*), relative clause (e.g. *who complimented the editor*), main clause (e.g. *revised the article*), and modifier (e.g. *for the newspaper*). The participant could view each region for as long as they liked, until pressing space to move onto the next region.

After the last region of the sentence, a question was presented which asked about the content of the sentence. Half of the questions probed the thematic relations of the relative clause (e.g., *Did the journalist compliment the editor?*), and half probed the main clause/modifier (e.g., *Was an article revised for a book?*). For each of these question types, the correct answers were 50/50 yes/no. Each of these categories were divided between the SR and OR conditions, such that they were balanced for question type and yes/no responses. Reaction times between the onset of each region and button press were recorded, as well as accuracy in the comprehension question. Four practice trials were presented first to familiarize participants with the task, using sentences distinct from the test material.

The outcome variables were either average sentence comprehension accuracy collapsing across canonical and noncanonical conditions (Acc), average relative clause (RC) response time collapsing across conditions (RT), or a structural disadvantage score (SDS) using either Acc or RT (accSDS, rtSDS). The SDS was calculated as the difference of the average scores of canonical (SR) compared with noncanonical (OR) conditions (e.g., SRRT - ORRT = rtSDS; ORacc - SRacc = accSDS) such that, for both rtSDS and accSDS, greater negative values indicate relatively greater difficulties on the noncanonical condition compared with the canonical one. This accounts for general accuracy or RT effects which may be related to age or other factors.

#### 2.2.2 National Institutes of Health list sorting working memory test and auditory–verbal short-term memory test

Here, we operationally define working memory as the ability to process information across modalities, keep that information in a short-term buffer, manipulate the information, and then keep the manipulated information in the same short-term buffer. To measure this ability, participants completed the National Institutes of Health List Sorting Working Memory Test (henceforth referred to as NIH Working Memory; NIH-WM (Tulsky et al., 2014; Weintraub et al., 2013)). NIH-WM is a sequencing task where participants must remember and then sort information. Participants are presented with a list of stimuli (e.g., illustrated images of animals or food), which are displayed one-by-one visually (word and picture) and auditorily for 2 seconds before moving onto the next item in the list. The task requires participants to remember each stimulus, order them from smallest to largest, and then recite the names of the stimuli in size order. There are two stages of the test, with the second stage having more complex directions. In the first part, the participant orders only one type of stimulus in a trial (e.g., animals) according to size, from smallest to largest. In the second part, the participant must order stimuli from two different categories (e.g., animals and foods). This second part of the test, a two-list task, requires participants to both “bin” the stimuli into one of two categories while also ordering each subgroup of stimuli in size order, from smallest to largest. List length increases by 1 with each correct response (up to a maximum of seven items). The task takes approximately 10–15 minutes to administer. The test is discontinued if two trials of the same item length are failed, or if the participant successfully completes the most difficult trial (two-list task, seven items to remember and order).

Here, we operationally define auditory–verbal STM as the ability to temporarily store and maintain auditory–verbal information for recall after a brief retention period. To measure this, participants completed the NIH Cognition Toolbox’s adaptation of the Rey auditory–verbal learning test (Rey, 1958; Weintraub et al., 2013), which is almost identical to the Rey AVLT but consists of three trials instead of five, no interference, and no delayed recall condition. Fifteen unrelated words are read aloud from the NIH toolbox application to the participant over three trials. After each trial, the participant must say back aloud, in any order, as many words as they can remember. The final raw score is the sum of the total number of items they were able to recall over the course of the three trials. Normative data for these tasks are available (Slotkin et al., 2012; Stricker et al., 2021).

#### 2.2.3 MRI acquisition and preprocessing

Participants underwent MRI scanning on a Siemens Trio 3T scanner with a 20-channel head coil. T1-weighted images were acquired using the following parameters: T1-weighted imaging (MP-RAGE) sequence with 1 mm isotropic voxels, 256 x 256 matrix size, 9° flip angle, and 92-slice sequence with repetition time (TR) = 2250 ms, inversion time (TI)=925 ms, and echo time (TE)=4.11 ms. Resting-state fMRI volumes (N = 427, duration = 10:30) were collected with the following parameters: echo planar imaging (EPI) sequence with 2.4 x 2.4 x 2.0 voxels, 216 x 216 x 120 matrix size, and interleaved ascending 50-slice sequence 20% slice gap and with TR = 1650 ms and TE = 35 ms.

T1-weighted (T1w) images were skull stripped and normalized to the MNI152 template using our publicly available *nii_preprocess* pipeline, relying on non-linear registration implemented in SPM12 (Penny et al., 2011). Segmentation of T1w images was performed with the Computational Anatomy Toolbox 12 (Gaser et al., 2024) for voxel-based morphometry and default settings to generate independent whole-brain volumetric estimates for gray matter, white matter, and cerebrospinal fluid (Ashburner, 2009). These maps were subsequently smoothed using an 8 mm full-width at half-maximum (FWHM) kernel. Volumes were collapsed over voxels within all gray matter regions in the Johns Hopkins University neuroanatomical atlas (Faria et al., 2012; Oishi et al., 2010). This procedure yielded a single average gray matter volume value for each ROI, in each participant.

Partial amplitude of low-frequency fluctuation was calculated using code described by Zou et al. (2008). This code was adapted to work with NIFTI images and is given as part of the nii_preprocess repository (https://github.com/neurolabusc/nii_preprocess). Briefly, we computed pALFF as the ratio of the power spectrum within the low-frequency band (0.01–0.08 Hz) to the power across the entire detectable frequency range (derived from each scan’s TR; TR = 1.65 s -> 0.303 Hz), and then normalized each voxel’s value by the mean within the brain mask.

## 3 Experimental Design and Statistical Analysis

### 3.1 Behavioral

Our primary aim was to examine whether working memory or STM mediated the relationship between age and sentence processing. Mediation analysis tests whether the effect of an independent variable (X) on a dependent variable (Y; i.e., X → Y) is partially explained by how X affects an intervening mediator variable (M) and how the intervening variable (M) affects the dependent variable (i.e., X → M → Y (Baron & Kenny, 1986; Hayes & Rockwood, 2017)). All behavioral statistics were conducted in Python 3 using Pingouin (Vallat, 2018).

A mediation model with three input variables (X, Y, M), such as ours, provides five paths. Path (a) M ~ X: linear relationship between M and X; Path (b) Y ~ M: linear relationship between M and Y; Path (c’) Direct Effect: relationship between X and Y that is not mediated by M; Path (ab) Indirect Effect: the strength of the mediating effect where M carries the influence of X to Y; Path (c) Total Effect: model that simultaneously uses both the direct and indirect effects to predict Y.

In the mediation models, we specified age as the independent variable (X) and working memory or STM as the mediator (M). Our dependent variable (Y) was either average sentence comprehension accuracy collapsing across canonical and noncanonical conditions (Acc), average relative clause response time collapsing across conditions (RT), or a structural disadvantage score (SDS) using either Acc or RT (accSDS, rtSDS). The SDS was the difference of the average scores of canonical (SR) compared with noncanonical (OR) conditions (e.g., SRRT - ORRT = rtSDS; ORacc - SRacc = accSDS) such that, for both rtSDS and accSDS, greater negative values indicate relatively greater difficulties on the noncanonical condition compared with the canonical one. Behavioral measures were z-transformed prior to analysis for interpretation of standardized regression coefficients. All mediation analyses included sex and level of education as nuisance covariates, and used a non-parametric bootstrapping method (n boot = 5000) to determine significance. We used a one-tailed hypothesis, with the prediction that older age would be associated with reduced memory capacity, which would, in turn, be associated with worse sentence processing.

### 3.2 ROI-based analyses

We focused our analysis on three main bilateral regions, with four subregions each (12 left-hemisphere and 12 right-hemisphere ROIs), which are broadly implicated in language and language-related processes (Binder & Desai, 2011; Binder et al., 2009; Desai & Riccardi, 2021; Hickok & Poeppel, 2007; Kemmerer, 2022). Parcellation used the Johns Hopkins University atlas (Faria et al., 2012). (1) *Frontal cortex*: inferior frontal gyrus pars triangularis (IFGtri), IFG pars opercularis (IFGoper), IFG pars orbitalis (IFGorb), and middle frontal gyrus (MFG); (2) *mid-to-anterior temporal lobe*: middle and superior temporal gyri (MTG; STG) and middle and superior temporal poles (MTGpole, STGpole); (3) *temporoparietal junction*: posterior middle and superior temporal gyri (pMTG, pSTG), supramarginal gyrus (SMG), and angular gyrus (AG).

**Fig. 1.**
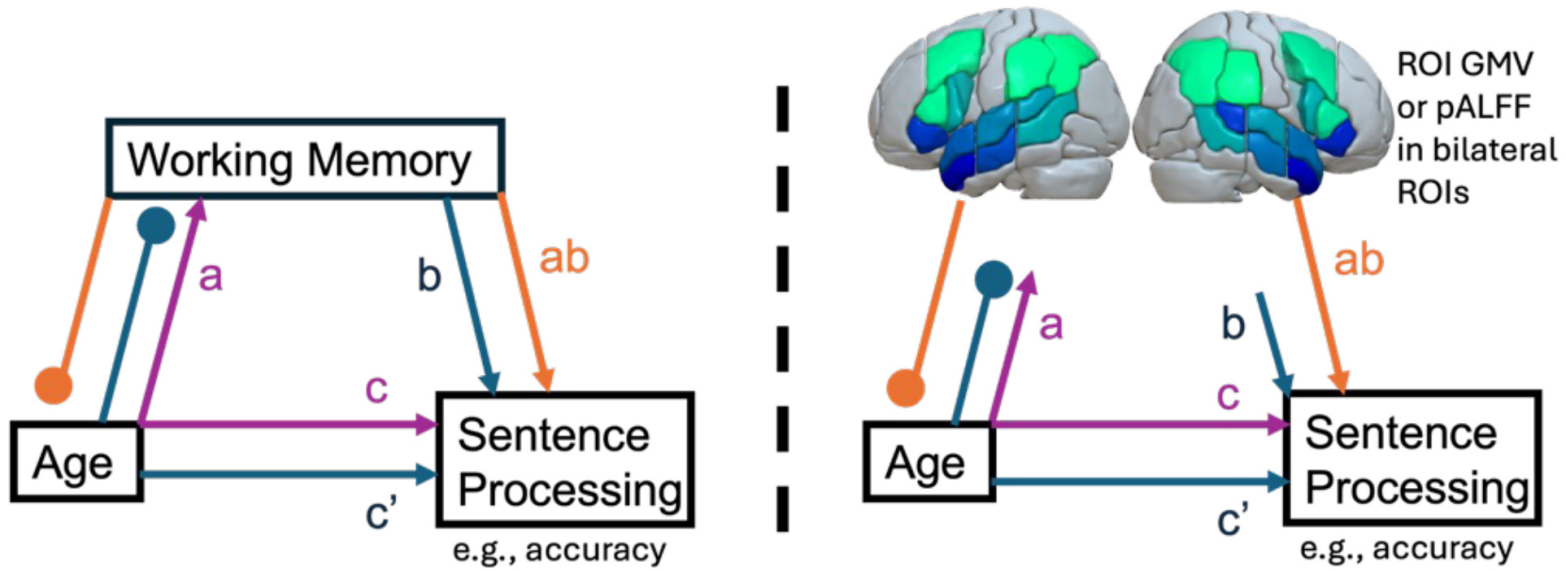
Path diagram showing the mediation analyses that were performed to understand the influence of age on sentence processing through reductions in working memory/STM (left) or region-wise brain volume or pALFF (right; 12 bilateral ROIs, color indicates mean GMV for each ROI; darker blue = less mean GMV, brighter green = greater mean GMV), while controlling for nuisance covariates. Path *a* assesses the effect of age on the mediator. Path *b* assesses the effect of the mediator on sentence processing. Path c assesses the total effect of age on sentence processing, combining direct and indirect effects. Path *c’* assesses the direct effect of age on sentence processing, independently of the indirect effect. Path *ab* assesses the indirect effect of age on sentence processing through the mediator.

Our in-house developed neuroimaging mediation toolbox uses the same statistical approach described above for behavioral variables (Fig. 1), where age is the independent variable (Y), sentence processing (Acc, RT, etc.) is the dependent variable (X), and GMV or pALFF in each prespecified ROI is the mediating variable (M). That is, age can be related to changes in brain structure/function or sentence processing difficulties, but only some brain areas might be expected to act as mediators between age and sentence processing. This would identify areas where preserved gray matter or pALFF relates to preserved sentence processing across the lifespan. Additionally, language-specific brain areas can be identified by including memory capacity in the model as a nuisance covariate, identifying areas where GMV or pALFF mediates age-related declines in sentence processing regardless of accompanying decline in working memory or STM. ROI mediation was run using the publicly available graphical Brain Association Toolbox (gBAT; https://github.com/alexteghipco/gBAT (Rangus et al., 2024)), developed by our group. The scripts leverage prior work done primarily by the Cognitive and Affective Neuroscience Lab (CANlab; https://github.com/canlab/MediationToolbox (Atlas et al., 2010; Chen et al., 2018; Geuter et al., 2020; Kenny et al., 2003; Shrout & Bolger, 2002; Wager et al., 2008; Wager, van Ast, et al., 2009; Wager, Waugh, et al., 2009; Woo et al., 2015).

In gBAT, we used mediation with robust ordinary least squares regression, nonparametric bias-corrected bootstrapped p-values, and a one-tailed hypothesis (alpha = .05). Total intracranial volume (TIV), sex, and education were included as nuisance covariates in all GMV models (TIV was not included in the pALFF-based models). All p-values were false discovery rate (FDR (Benjamini & Hochberg, 1995)) corrected, with left and right hemisphere ROIs being FDR corrected separately within each imaging modality. This was done because pALFF and GMV represent distinct neurobiological phenomena, and because we hypothesized different roles for the structure and function of the left and right hemispheres in mediating age-related declines. Reported p-values in the ROI analysis specifically reflect the indirect effect (i.e., the effect of age on the dependent variable that is mediated by regional GMV or pALFF).

We conducted four main analyses. First, using traditional correlational methods (not mediation), we examined where GMV and pALFF were correlated with age. Second, we investigated whether ROI GMV or pALFF mediated the relationship between age and overall sentence comprehension (Acc) or relative clause reaction times (RT), with and without working memory and STM as a nuisance covariate. Third, we investigated whether ROI GMV or pALFF mediated the relationship between age and Acc SDS or RT SDS, again with and without working memory and STM as a nuisance covariate. Finally, we sought areas where GMV or pALFF mediates the relationship between increased age and decreased working memory or STM.

## 4 Results

### 4.1 Behavioral

Behavioral results are organized according to the dependent variable, with working memory reported first and the STM analyses reported after (Table 2). P-values reflect one-tailed hypothesis testing. The relationship between age and working memory is a “path” in all our models, so we report it once here: increased age was significantly associated with decreased working memory (β = -.61, p < .001) and decreased STM (β = -.54, p < .001).

**Table 2.** Mediation results where the mediator is working memory (top) or STM (bottom), with Y variables being indicated in the columns.

X: Age				
Mediator (M): NIH Toolbox WM				
Y:	Overall Acc	accSDS	Overall RT	rtSDS
M ~ Y	**β** **=** **0.35** **p** **<** **.001**	**β** **=** **0.12** **p** **=** **.05**	**β** **=** **-0.22** **p** **=** **.001**	**β** **=** **0.14** **p** **=** **.028**
Total	**β** **=** **-0.16** **p** **=** **.017**	β = -0.1 p = .095 ~	**β** **=** **0.27** **p** **<** **.001**	**β** **=** **-0.17** **p** **=** **.008**
Direct (X→Y)	β = 0.09 p = .135	β = -0.04 p = .35	**β** **=** **0.21** **p** **=** **.01**	β = -0.14 p = .06 ~
Indirect (X→M→Y)	**β** **=** **-0.25** **p** **<** **.001**	β = -0.06 p = .17	β = 0.05 p = .15	β = -0.03 p = .26

Mediator (M): NIH Auditory Verbal Learning Test				
Y:	Overall Acc	accSDS	Overall RT	rtSDS
M ~ Y	**β** **=** **0.25** **p** **<** **.001**	**β** **=** **0.14** **p** **=** **.033**	**β** **=** **-0.22** **p** **=** **.001**	β = 0.09 p = .11
Total	**β** **=** **-0.16** **p** **=** **.017**	β = -0.1 p = .095 ~	**β** **=** **0.27** **p** **<** **.001**	**β** **=** **-0.17** **p** **<** **.001**
Direct (X→Y)	β = -0.03 p = .36	β = -0.03 p = .35	**β** **=** **0.21** **p** **=** **.01**	**β** **=** **-0.18** **p** **=** **.02**
Indirect (X→M→Y)	**β** **=** **-0.13** **p** **=** **.005**	β = -0.06 p = .075 ~	β = 0.06 p = .12	β = 0.01 p = .47

Bold indicates p < .05, ~ indicates p < .1.

Acc: accuracy; RT: response times; SDS: structural disadvantage score (difference between canonical and noncanonical conditions).

#### 4.1.1 Overall accuracy

**Fig. 2.**
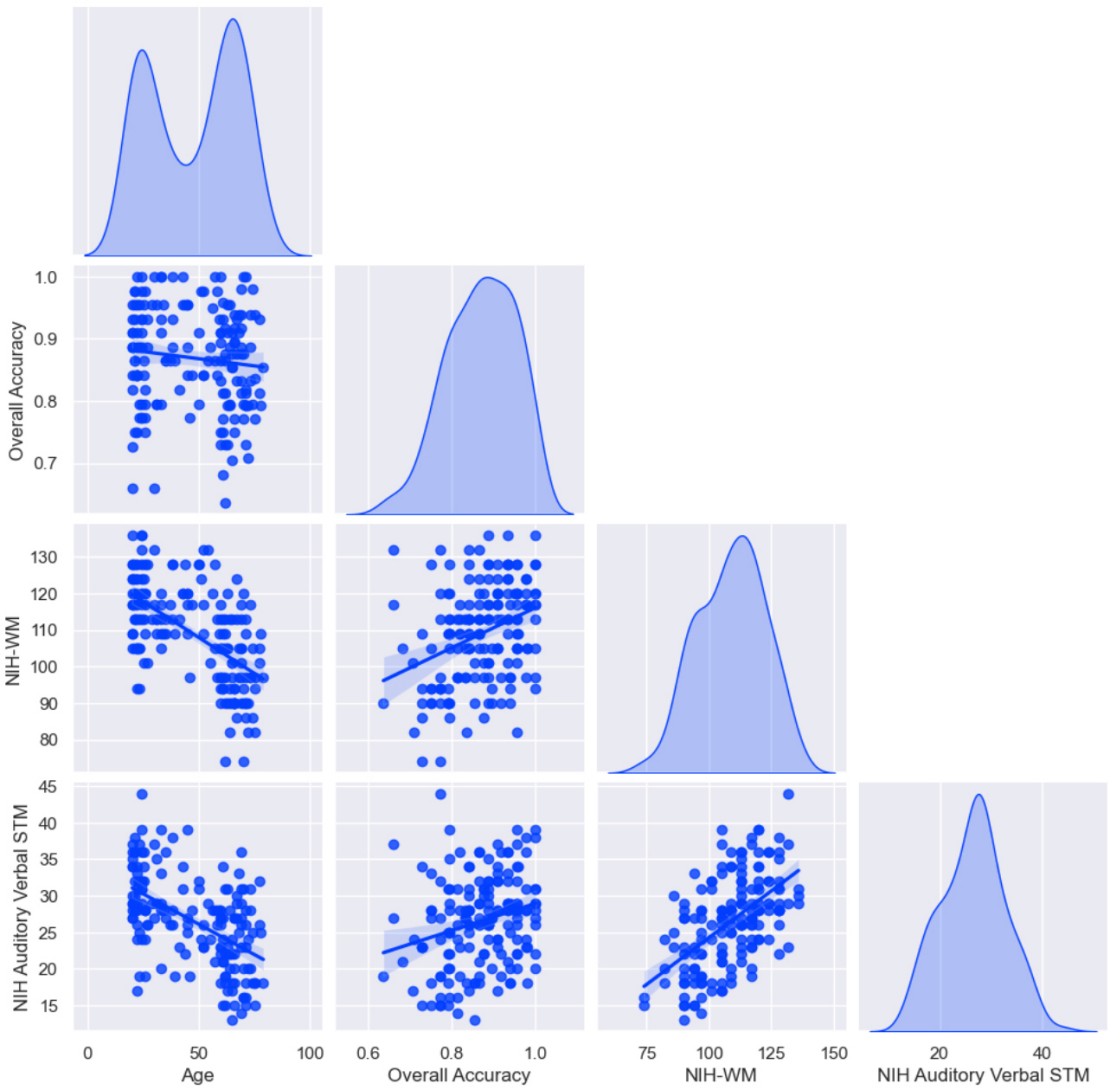
Relationships between age, sentence comprehension accuracy, NIH Working Memory, and NIH Auditory Verbal STM. NIH WM and NIH STM both mediated the relationship between age and accuracy, where preserved memory capacity across the lifespan relates to better comprehension accuracy. Additional plots between age and behavioral variables are given in the Supplementary Materials.

Older age was correlated with worse Acc (r = -.16, p = .015). Increased working memory was associated with better Acc (β = .35, p < .001). The total effect of age and working memory on Acc was significant (β = -.16, p = .017). The direct effect of age on Acc was not significant (β = .1, p = .135). The indirect effect of working memory on Acc was significant, indicating that working memory mediates age-related decline in Acc (β = -.25, p < .001; Fig. 2).

Increased STM was associated with better Acc (β = 0.25, p < .001). The total effect of age and STM on Acc was significant (β = -0.16, p = .017). There was no direct effect of age on Acc (β = -0.03, p=.36), but a significant indirect effect of STM on Acc (β = -0.13, p = .005), indicating a complete mediation.

#### 4.1.2 accSDS

Older age was not significantly correlated with more negative accSDS (e.g., relatively greater difficulty on the OR sentences; r = -.1, p = .095). Lower working memory was associated with more negative accSDS (β = 0.12, p = .05). The total effect of age and working memory on accSDS was not significant (β = -0.1, p = .095). There was not a significant direct effect of age on accSDS (β = -0.04, p = .34), and there was not an indirect effect of working memory on accSDS (β = -0.06, p = .17).

Decreased STM was associated with more negative accSDS (β = 0.14, p = .033). The total effect of age and STM on accSDS was not significant (β = -0.1, p = .095). There was no direct effect of age on Acc (β = -0.03, p = .35), and a trending but insignificant indirect effect of STM on accSDS (β = -0.06, p = .075).

#### 4.1.3 Overall RC RT

Older age was correlated with longer RT (r = .27, p < .001). Lower working memory was associated with longer RT (β = -0.23, p = .001). There was a significant total effect of age and working memory on RT (β = 0.27, p < .001). There was a significant direct effect of age on RT (β = 0.21, p = .01). There was not a significant indirect effect of working memory on RT, indicating that working memory does not significantly mediate age-related decline in sentence processing speed (β = 0.06, p = .16).

Decreased STM was associated with longer RT (β = -0.22, p = .001). There was a significant total effect of age and STM on RT (β = 0.27, p < .001). There was a significant direct effect of age on RT (β = 0.21, p = .01). There was not a significant indirect effect of STM on RT (β = 0.06, p = .11).

#### 4.1.4 rtSDS

Older age was correlated with more negative rtSDS, indicating relatively greater difficulty for older adults on the OR (noncanonical) sentences (r = -.18, p = .008). Lower working memory was also associated with more negative rtSDS (β = 0.14, p = .028). There was a significant total effect of age and working memory on rtSDS (b = -.005, p = .008). There was not a significant direct effect of age on rtSDS (b = -.004, p = .06). There was not a significant indirect effect of working memory on RT (p = .26).

Decreased STM was not associated with more negative rtSDS (p = .11). There was a significant total effect of age and STM on rtSDS (β = -0.17, p =.008). There was a significant direct effect of age on rtSDS (β = -0.18, p = .02), but no mediating effect of STM on rtSDS (β = 0.01, p = .47).

### 4.2 ROI-based GMV and pALFF mediation

First, using a traditional correlational approach, we evaluated areas where GMV or pALFF was associated with age, controlling for sex and education (and TIV in GMV analysis). Then, using mediation analysis, we assessed the significant indirect effects where GMV or pALFF of the individual ROIs were mediators between age and the dependent variable. For ease of reporting and interpretability, we only report indirect effects. When the indirect effects are of primary interest (as they are here), it is appropriate to report and interpret significant indirect effects regardless of the significance of total effects (Hayes, 2009, 2017; Shrout & Bolger, 2002). We also examined where GMV or pALFF were mediators between age and working memory or STM. P-values are FDR corrected, unless otherwise specified.

#### 4.2.1 Age, GMV, and pALFF

**Fig. 3.**
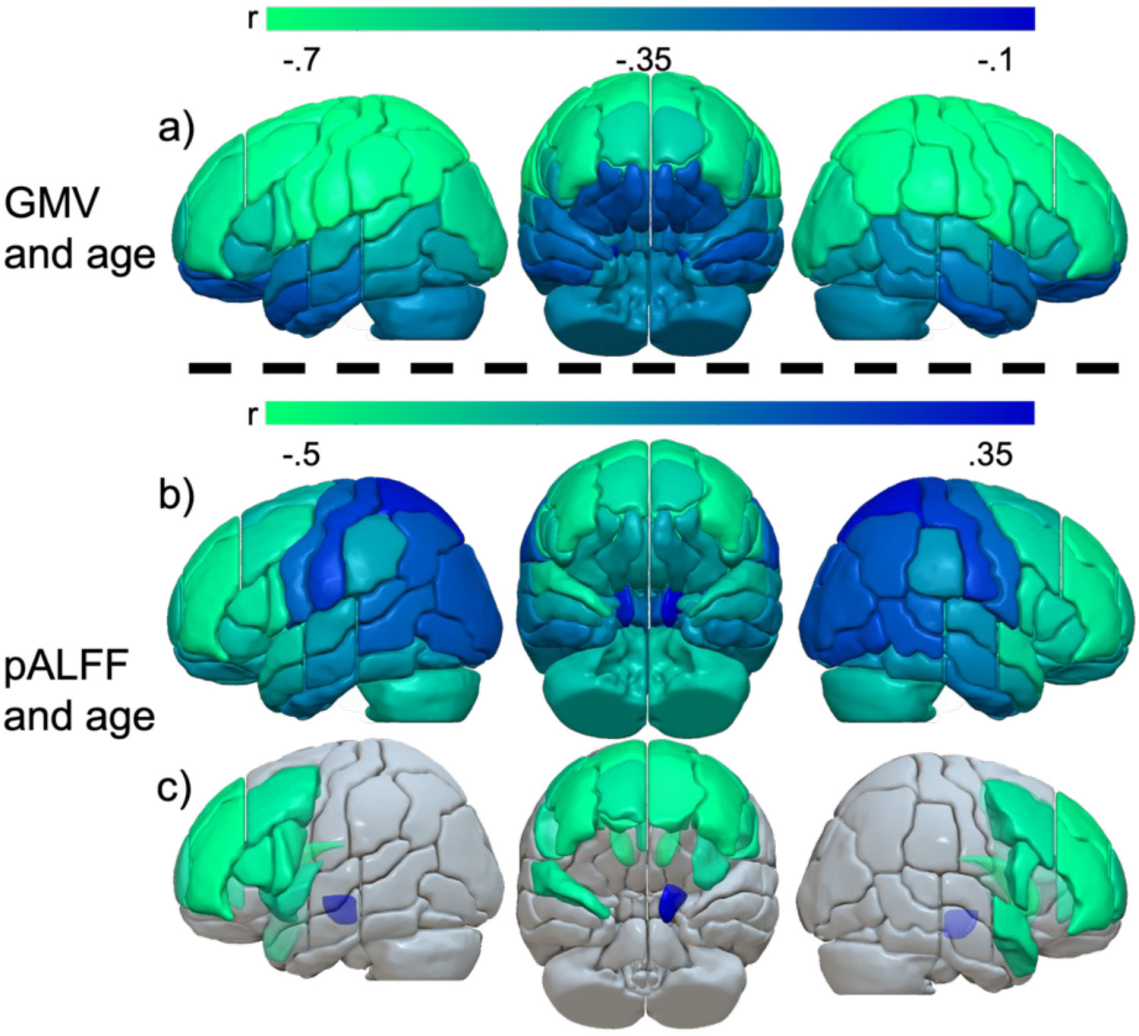
GMV (a) and pALFF (b, c) correlations with age, controlling for sex, education, and TIV (for GMV). Brighter green indicates stronger negative correlations with age. (a) areas where GMV was significantly correlated with age (one-tailed FDR p < .05), (b) pALFF and age correlation coefficients, (c) areas where pALFF was significantly correlated with age (two-tailed FDR p < .05).

In every gray matter region in the JHU atlas except for bilateral entorhinal cortex, nucleus accumbens, and red nucleus, GMV was significantly negatively correlated with age (all p’s < .05; correlation coefficients shown in Fig. 3). pALFF was significantly negatively correlated with age in bilateral frontal and right anterior temporal areas, and it was significantly positively correlated with the left cerebral peduncle.

#### 4.2.2 GMV

**Fig. 4.**
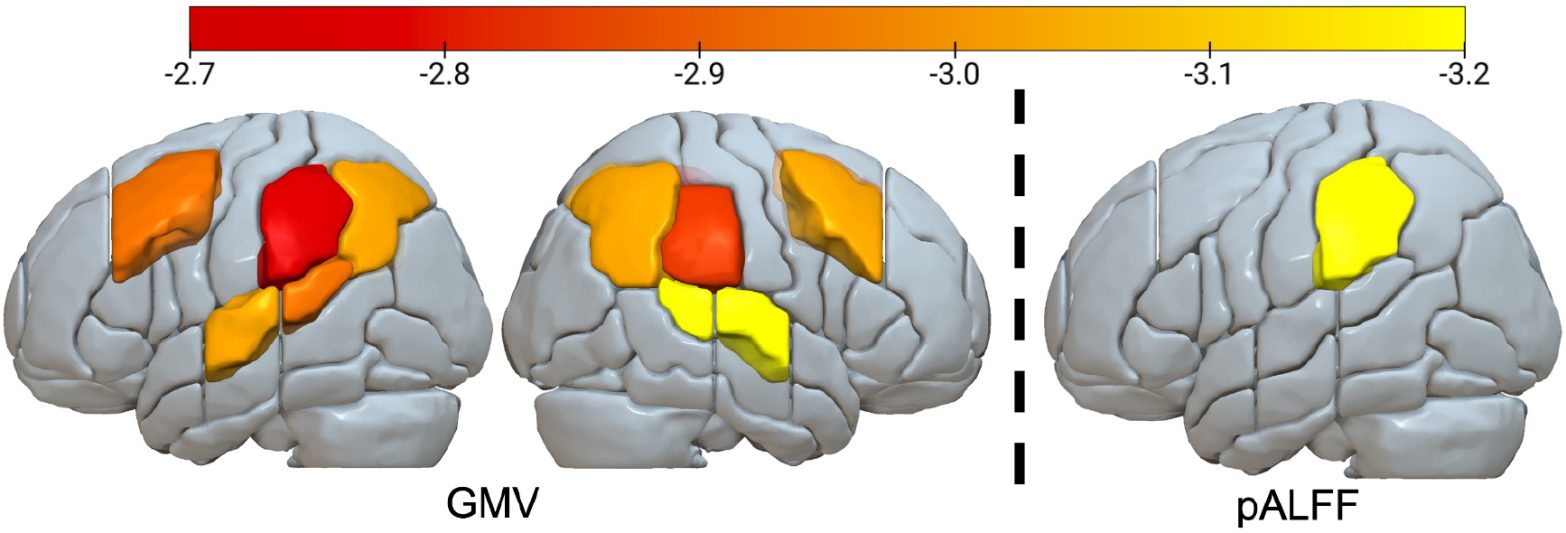
Areas where GMV (left) and pALFF (right) significantly mediated the relationship between age and rtSDS (FDR p’s < .05). GMV results survived while simultaneously controlling for sex, education, TIV, working memory, and auditory–verbal STM. pALFF results survived when controlling for working memory or auditory–verbal STM separately, but not simultaneously. Colors indicate the t-stat of the indirect effect, where lower GMV or pALFF is associated with greater relative response times in OR (noncanonical) sentences compared with SR sentences. Brighter colors indicate stronger negative effects.

GMV in the bilateral MFG, SMG, AG, STG, and pSTG significantly mediated the relationship between age and rtSDS (all p’s < .05; Fig. 4) controlling for both working memory and STM. GMV did not significantly mediate the relationships between age and the other dependent variables of interest (Overall Acc, Overall RT, or accSDS).

#### 4.2.3 pALFF

**Fig. 5.**
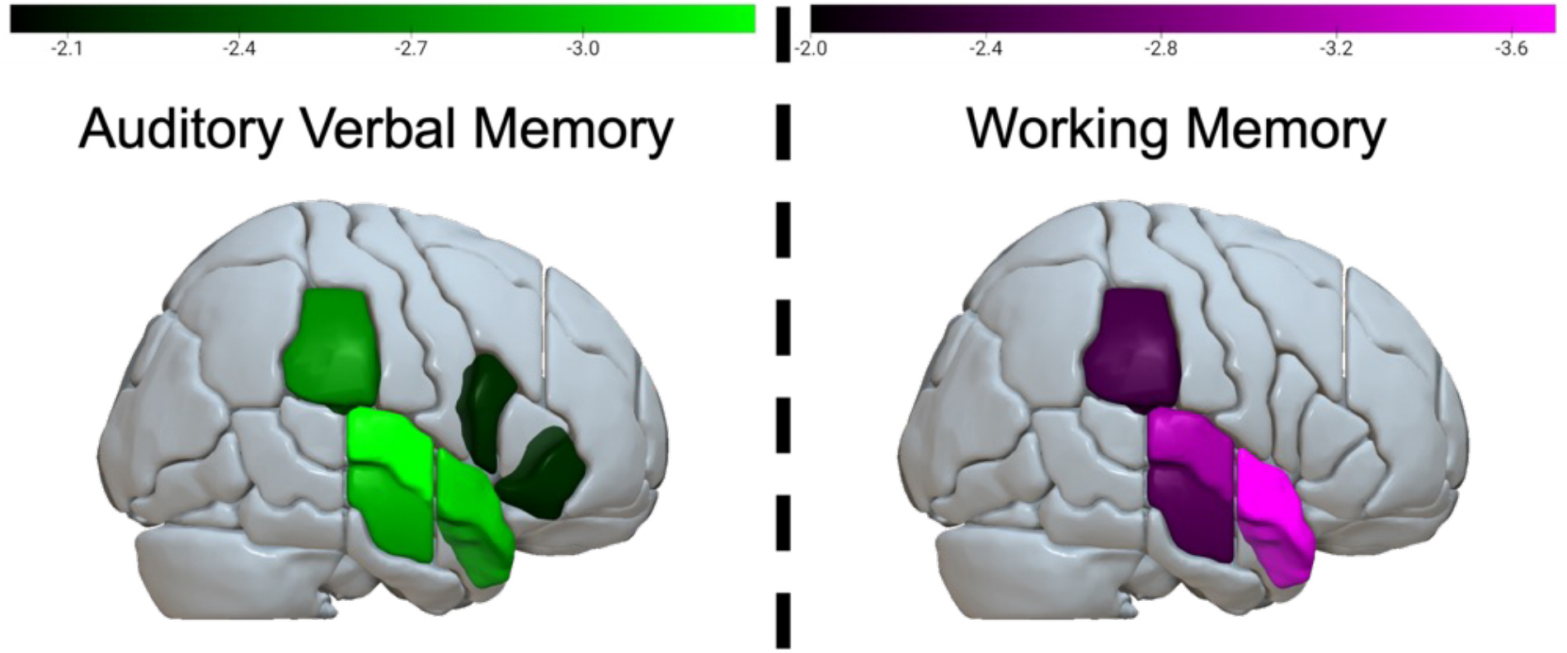
Areas where pALFF significantly mediated the relationship between age and auditory–verbal STM (left) and working memory (right), after controlling for the other memory measure. Areas significant in both models indicate shared neural substrates underlying both WM and STM. The indirect effect indicated that lower activity was associated with worse memory capacity. Colors indicate the t-stat of the significant indirect effects after FDR correction, with brighter colors showing stronger negative effects.

pALFF in the left SMG significantly mediated the relationship between age and RT SDS when controlling for WM or STM individually (p = .02; Fig. 4), but this relationship was not significant when controlling for both WM and STM simultaneously. pALFF in the right IFGoper, IFGtri, SMG, STG, STGpole, and MTG mediated the relationship between age and STM (p’s < .05; Fig. 5), after controlling for WM. pALFF in the right SMG, STG, STGpole, and MTG mediated the relationship between age and WM (p’s < .05), after controlling for STM. Areas significant in both models indicate shared neural substrates underlying both WM and STM. GMV did not significantly mediate the relationship between age and WM or STM.

## 5 Discussion

Here, we examined relationships between age, working memory/STM, sentence processing, and brain structure and function using a novel mediation approach. Working memory and verbal STM capacity mediated the relationship between age and offline SPR comprehension accuracy. Age, and not memory capacity, was the most important factor for online processing speed of the relative clause. Bilateral GMV in posterior temporal/inferior parietal areas and pALFF in the left SMG mediated age-related online processing speed for OR compared with SR conditions, controlling for memory capacity. Age-related decline in memory capacity was mediated by pALFF in right hemisphere frontotemporoparietal regions.

### 5.1 How does gray matter volume and resting-state pALFF change with age?

GMV throughout the brain gradually decreases with age, but certain brain areas are more robust against volumetric decline (Hedman et al., 2012; Oschwald et al., 2019). When compared with frontal or dorsal areas, ventral and medial temporal structures tend to be resilient against atrophy (Good et al., 2001; Resnick et al., 2003; Smith et al., 2007; Storsve et al., 2014; Ziegler et al., 2012). Instead, these areas undergo decline mainly in advanced age (e.g., > 70 years) or in individuals with impaired cognitive status or neurodegenerative pathologies (Holbrook et al., 2020; Igarashi, 2023; Olsen et al., 2017; Tamnes et al., 2013), which are underrepresented (or not present) in our cohort. Our results align with this body of work and add to it, showing that increased age was related to decreased GMV throughout the brain (especially parietal and frontal lobes), with the weakest age-related effects being seen in the ventral temporal lobes and entorhinal cortex.

Although pALFF is a desirable rsFMRI measure to use in aging because of its robustness against physiological artifacts (Zou et al., 2008), most previous studies of healthy aging have used functional connectivity (Dennis & Thompson, 2014; Liem et al., 2021). pALFF and functional connectivity are partially orthogonal. Decreases in pALFF (regional intrinsic activation) do not necessarily indicate decreases in time-locked activation between two areas (connectivity). Previous studies using pALFF identified bilateral frontal and anterior cingulate cortex decreases with increased age (Damoiseaux et al., 2008; Hu et al., 2014). Preserved pALFF in bilateral frontal areas may also be protective against cognitive decline (Han et al., 2011; Lee & Hsieh, 2017; Lin et al., 2016; Rafiq et al., 2022; Ren et al., 2015). In the current study, increased age was significantly related to decreased pALFF in bilateral frontal association cortices, both laterally and medially. Bilateral occipital and parietal areas displayed increased (but not significant) pALFF with increased age. As discussed below, preserved pALFF in right hemisphere inferior frontal and temporoparietal regions was associated with better memory capacity across the lifespan. The current results add to the body of literature examining how brain structure and function changes in increased age, and how those changes affect cognition.

### 5.2 Does memory capacity mediate age-related declines in sentence processing?

Linear relationships between age, memory capacity, and sentence processing (paths *a*, *b*, and *c* in our mediation analyses) revealed that (1) older age was associated with worse offline SPR comprehension, overall RC RT, and rtSDS, (2) older age was related to decreased working memory and verbal STM, and (3) working memory and verbal STM were both significantly related to overall comprehension accuracy, overall RC RT, and accSDS, but not rtSDS. This is consistent with prior work (Caplan & Waters, 2013; Gilardone et al., 2024; Grossman et al., 2002; Lewis et al., 2006; Salis, 2011; Small et al., 2000; Waters et al., 2016).

More innovatively, examination of the direct and indirect effects (paths *c’* and *ab*), which are unique to mediation analyses, revealed the independent contributions of age on sentence processing and the mediating effect of memory capacity on age-related declines in sentence processing. The primary direct effect of age was on online measures (overall RT and rtSDS). However, WM and verbal STM had significant mediating effects for offline comprehension accuracy, but no effects on online response times.

The results for overall accuracy and RC RT expand upon the previously discussed work examining the relationships between age, memory capacity, and sentence processing. For example, Van der Linden et al. (1999) found that working memory mediated the relationship between age and offline text comprehension, sentence recall, and story recall. DeDe et al. (2004) found that verbal working memory, but not age, was important for offline sentence comprehension, while age, but not verbal working memory, is most related to online sentence processing. Our results closely align with those findings. Working memory and verbal STM were significant mediators of age-related declines in the offline comprehension task, while age had a significant direct effect on online RC RT, with no mediating effects of memory capacity. A possible interpretation for the offline comprehension results is that the comprehension task itself requires memory encoding and retrieval, effectively making it a “memory task.” An alternative interpretation is that memory capacity aids in thematic role assignments that allow for comprehension questions to be answered successfully (e.g., “who did what to whom” (Finocchiaro et al., 2015; Thothathiri et al., 2018)). These hypotheses await further testing.

Compared with overall accuracy and RT, results for the structural difference scores (accSDS, rtSDS) were somewhat unexpected. Neither working memory nor verbal STM were significant mediators of age-related decline in noncanonical compared with canonical syntactic structures. Direct effects (path *c’*) revealed that age affected rtSDS but not comprehension (accSDS). That is, older adults performed worse for noncanonical conditions compared with canonical ones during online sentence processing (rtSDS), but these differences are less apparent in offline tasks that allow for delayed processing (accSDS). Multiple cognitive models exist to explain the nature of the relationship between working memory or verbal STM capacity and syntax in aging (Caplan & Waters, 2013; Carpenter & Just, 2013; Gathercole & Baddeley, 2014). The capacity theory posits that overall working memory capacity allows for more cognitive resources to be allocated when encountering complex syntactic structures (Carpenter & Just, 2013; Just & Carpenter, 1992), while other models posit that there may be a separate system for syntactic parsing that is at least partially dissociable from domain-general working memory abilities (Caplan & Waters, 1999, 2013; Waters & Caplan, 1996a; Waters et al., 2016). Our results provide partial support for models suggesting that increased memory capacity is beneficial for sentence processing. Preservation of memory capacity across the lifespan supported offline comprehension but not online processing speed. Further, memory capacity did not significantly mediate the differences in OR compared with SR structures in the task employed here, suggesting that either language-specific processes or alternative domain-general resources (e.g., fluid intelligence (Cattell, 1963)) underlie age-related difficulties in more complex syntactic structures.

### 5.3 Does GMV or pALFF mediate sentence processing and memory capacity across the lifespan?

ROI-based mediation analyses revealed that bilateral temporoparietal and middle frontal GMV mediated the relationship between age and online rtSDS. Reduced GMV in these areas was associated with slower processing speed at the relative clause for OR (noncanonical) compared with SR (canonical) sentences. This relationship survived after simultaneously controlling for both working memory and verbal STM capacity. Lower pALFF specifically in the left SMG was also related to slower processing speed at the relative clause for OR relative to SR sentences, controlling for working memory or verbal STM individually, but not simultaneously. Preserved right hemisphere pALFF in temporoparietal and inferior frontal regions mediated age-related declines in memory capacity. GMV was not a significant mediator of the memory tasks used here.

p/STG, SMG, MFG, and AG are well-established parts of the “language cortex” (Desai & Riccardi, 2021; Kemmerer, 2022). For sentence comprehension specifically, functional neuroimaging meta-analyses have identified these areas as being especially involved in the comprehension of complex syntactic structures (Hagoort & Indefrey, 2014; Meyer & Friederici, 2016). Damage or disruption of the temporoparietal junction in post-stroke aphasia is associated with impairments in sentence comprehension, especially for noncanonical structures (Caplan et al., 1996; den Ouden et al., 2019; Fridriksson et al., 2018; Kristinsson et al., 2020; Magnusdottir et al., 2013; Matchin et al., 2023; Riccardi et al., 2022; Rogalsky et al., 2018). Indeed, the brain regions related to rtSDS in the current study display striking overlap with the body of lesion-symptom mapping evidence in post-stroke aphasia. In neurologically intact aging, sentence processing in older adults tends to be associated with altered activation in bilateral frontal areas or right hemisphere language homologues compared with younger adults, although it is debated whether these activation patterns are beneficial or maladaptive (Antonenko et al., 2013; Davis et al., 2014; Peelle et al., 2010; Pistono et al., 2021; Rafiq et al., 2022; Tyler et al., 2010). The relationship between normal age-related brain atrophy and sentence processing is also unclear, with some studies suggesting that cognitive or functional changes compensate for structural atrophy (Campbell et al., 2016; Pistono et al., 2021; Tyler et al., 2010). In this context, our findings provide novel insights about neurobiological models of sentence processing in aging.

(1) A role for left posterior temporal/inferior parietal regions in complex sentence processing was supported. Despite previous research implicating these areas in memory processes that support sentence comprehension (Deschamps et al., 2014; Grasby et al., 1993; Guidali et al., 2019; Leff et al., 2009; Newhart et al., 2012; Park et al., 2011; Seghier, 2013; Vatansever et al., 2017), we suggest that the structural integrity of regions within the TPJ is supportive of complex sentence processing in a way that goes beyond memory capacity, as measured by the memory tasks used here. The mediating effect of GMV in these regions survived after simultaneously controlling for both working memory and verbal STM. Further, the effects were seen in the rtSDS condition, suggesting that the structural integrity of these areas is supportive specifically for noncanonical compared with canonical syntactic structures. These areas may support on-line hierarchical structure building or other syntactic processes (Matchin & Hickok, 2020; Walenski et al., 2019), which show themselves as slower response times when encountering the relative clause in difficult sentence structures.

Lower pALFF in the left SMG was related to slower processing speed at the relative clause for OR compared with SR sentences, controlling for working memory or verbal STM individually, but not simultaneously. This finding can be synthesized with the above GMV results when considering the SMG’s established role in memory-related processes (including phonological, working memory, and verbal STM) that aid in language comprehension (Binder, 2017; Deschamps et al., 2014; Guidali et al., 2019; Oberhuber et al., 2016). We propose that, within the left TPJ, preserved activity in the SMG at least partially aids complex sentence processing via memory-related functions. The specific processes subserved by the SMG may differ between individuals (Booth et al., 2002; Welcome & Joanisse, 2012), depend on cognitive strategies employed during the SPR task (Choi et al., 2017; Stine-Morrow et al., 1996; Zhang et al., 2022), and likely differ within the SMG itself, which has been shown to have at least four functionally distinct subregions that aid word processing (Oberhuber et al., 2016).

(2) The importance of right hemisphere structural integrity in sentence processing across the lifespan was supported. In many neurobiological models of language processing, the role of the right hemisphere in language processing remains unclear or underspecified (Desai & Riccardi, 2021), due to language being largely left lateralized. However, studies increasingly show that the right hemisphere might play an important supporting role for language. In aphasia following left unilateral stroke, functional connectivity and activity in right hemisphere temporoparietal regions such as the angular gyrus can be predictive of language comprehension and production (Cocquyt et al., 2017; Klingbeil et al., 2019; Riccardi et al., 2019, 2020; Riccardi, Schwen Blackett, et al., 2024;; Riccardi, Zhao, et al., 2024). A recent review of fMRI studies in neurologically intact adults suggested that there is an interaction between language and task difficulty, wherein right hemisphere areas such as the AG, SMG, and IFC mainly contribute to difficult language tasks that have increased processing demands (Deldar et al., 2021). Indeed, activity in the right hemisphere has been related to language task performance even in healthy adults (Lidzba et al., 2011; Powell et al., 2012; van Ettinger-Veenstra et al., 2010).

While this is typically thought of in the context of right hemisphere brain *function*, here we find novel evidence that implicates right hemisphere brain *structure* in mediating age-related sentence processing declines. The right hemisphere GMV results observed here cannot be attributed to memory capacity (as measured by the tasks used here), although we cannot rule out other domain-general mechanisms. One could argue that, because age-related atrophy tends to be symmetrical, the current study cannot necessarily disentangle unique contributions of the left versus right hemisphere structural integrity. However, as seen in the pALFF results, unilateral mediation effects can be detected even in the case of equivalent, hemispherically symmetric values, suggesting that the structural integrity of right hemisphere regions has a mechanistic influence on sentence processing.

(3) The importance of right hemisphere function in memory capacity across the lifespan was supported. Preserved pALFF in the right SMG, STG, MTG, and the pole of the superior temporal gyrus (STGpole) was related to better working memory and verbal STM capacity across the lifespan. pALFF in the right inferior frontal gyrus pars opercularis and pars triangularis was related to verbal STM, but not working memory capacity. While the specific areas identified are not necessarily surprising, the right laterality of the effects provides valuable insights about the neurobiology of aging. These areas are involved in the phonological and articulatory loop during the maintenance and manipulation of items in memory, which would be expected to be largely left lateralized because of the word-based memory tasks used here (Chai et al., 2018; Deldar et al., 2021; Marvel & Desmond, 2012; McGettigan et al., 2011).

The observed benefits of increased right hemisphere brain activity on memory capacity can provide insights into two competing theoretical frameworks for the neurobiology of aging. Some models predict that, as people age, reliance on the right hemisphere or domain-general regions should increase relative to younger adults (Hemispheric Asymmetry Reduction in Older Adults (Cabeza, 2002; Dolcos et al., 2002); Compensation-related Utilization of Neural Circuits Hypothesis (Kang et al., 2021; Reuter-Lorenz & Cappell, 2008)). Other work has found evidence against the hypothesis that older adults undergo reorganization of task-related brain networks (Campbell et al., 2016; Henson, 2006; Morcom & Johnson, 2015). Our results provide broad support for models predicting that memory performance in older adults is related to right hemisphere brain function.

(4) With the caveat being that negative results should not be strongly interpreted, the influence of structural integrity of the inferior frontal cortex in sentence processing across the lifespan was not supported. The specific role of the IFC in language and sentence processing remains contentious. Hypotheses include that the IFC is involved in hierarchical structure building or syntax-specific processes (Friederici, 2018; Grodzinsky & Santi, 2008; Hagoort, 2014; Hagoort & Indefrey, 2014; Meyer & Friederici, 2016; Zaccarella et al., 2017), or that it supports memory or other domain-general functions (Hickok, 2000; Matchin & Hickok, 2020; Rogalsky & Hickok, 2011; Rogalsky et al., 2008). Here, IFC structure and function were not related to any of the sentence processing measures. However, our neuroimaging measures were offline, as opposed to task-based measures. It is possible that IFC activation would have been observed if the task was completed in the scanner. Another hypothesis is that the IFC is especially related to syntactic processes during production (Chang et al., 2018; den Ouden et al., 2019; Giglio et al., 2022; Matchin & Hickok, 2020), and less so during comprehension, which would not be detected in the receptive sentence task used here.

### 5.4 Limitations and future directions

Our age distribution is bimodal, with a relatively small number of participants aged 30–50 years compared with those in younger and older ages. While our nonparametric statistical methods are robust against violations of normality, it would be ideal to have an even distribution of participants throughout the entire age range. Because the Aging Brain Cohort project focuses on neurologically intact aging broadly, as opposed to reading/syntax specifically, participants were not screened or excluded prior to analysis for a history of reading disorders. This may influence results, although our outlier removal procedure ensured that participants with reading RTs and accuracies greater than 3 standard deviations away from the mean were excluded before analysis.

Each participant saw both the SR and OR versions of each sentence. Although OR and SR sentences were randomly presented to each participant in a single continuous block to minimize the risk of priming or practice effects at a group level, we cannot rule out the possibility of these effects within individual participants. There is evidence that older adults employ different reading strategies than younger adults and that these strategies may change depending on the task demands (Choi et al., 2017; Stine-Morrow et al., 1996; Zhang et al., 2022). Although our task instructions encouraged all participants to progress as quickly as they felt comfortable with, we cannot rule out the possibility that older adults may have perceived the task as more of a “memory test,” thereby taking extra time during each sentence (n.b., this strategy would be expected to be employed for both SR and OR conditions, meaning that individual differences in reading strategy would have less of an impact on the structural disadvantage scores, which were the focus of our analyses).

The behavioral tasks and neuroimaging were conducted separately. While offline neuroimaging measures are powerful tools for understanding the neurobiology of aging, language, and pathologies (Ferreira & Busatto, 2013; Ferreira et al., 2011; Lerch et al., 2017; Smitha et al., 2017; van den Heuvel & Hulshoff Pol, 2010), it is possible that different neural substrates would have been identified in a task-based neuroimaging context. The SPR measures comprehension and processing speed, but not production. We cannot make claims about syntactic processes during sentence production, which overlap with, but sometimes dissociate from, the processes involved in sentence comprehension (Desai & Riccardi, 2021; Lukic et al., 2021; Matchin & Hickok, 2020; Walenski et al., 2019).

The NIH-WM and NIH Auditory Verbal Learning Tests were chosen at the inception of the ABC@USC project for their brevity, availability of normative data, strong test–retest reliability, good convergent validity with other measures intended for the same cognitive constructs, and sensitivity to cognitive change across the lifespan (Slotkin et al., 2012; Stricker et al., 2021; Tulsky et al., 2014; Weintraub et al., 2013). However, working memory and auditory–verbal STM are complex operations with distinct subprocesses. The NIH toolbox tasks provide broad indices of these constructs rather than fine-grained measures of specific theoretical components. This approach sacrifices some construct precision in favor of increased standardization and generalizability across studies, so some caution is warranted when comparing results with prior work that used more specialized memory tasks. Both tasks contain verbal/lexical-semantic components. To more “purely” isolate memory processes, a non-linguistic memory task would be ideal to include, but is unavailable in the ABC@USC project. Relatedly, although our working memory measure was accuracy based and our models accounted for general slowing by incorporating the difference score metric, it remains possible that age-related changes in processing speed or executive control contribute to the observed associations. Future studies incorporating multiple executive and speed measures could further disentangle these influences.

## 6 Conclusions

Here, using a novel ROI-based mediation approach, we examined relationships between age, working memory/STM, sentence processing, and the brain in a large cohort of neurologically intact adults. Behaviorally, we found that working memory and verbal STM capacity completely mediated the relationship between age and comprehension accuracy in the self-paced reading task. Age, and not memory capacity, was the most important factor for processing speed of the relative clause. Neuroanatomically, we found that bilateral GMV in posterior temporal/inferior parietal areas and pALFF in the left SMG mediated age-related processing speed for OR compared with SR conditions, even after controlling for memory capacity, suggesting that these areas support processing complex syntactic structures across the lifespan. Age-related decline in memory capacity was mediated by pALFF in right hemisphere frontotemporoparietal regions, aligning with neurobiological models of aging that predict that right hemisphere activity can be a compensatory strategy in old age.

## Supplementary Material

Supplementary Material

## Data Availability

Behavioral data, stimuli, and scripts are given here: https://osf.io/jwubc/. Brain mediation software is given at https://github.com/alexteghipco/gBAT. Data requests for brain imaging data can be submitted to: https://abc.sc.edu/abc-repository-data-requests/.

## Footnotes

1 Note that this is a retrospective analysis of data collected as part of a larger, ongoing study of brain health, genetics, and lifestyle. Task-based fMRI of the SPR or memory measures are not part of the testing battery, hence the use of resting-state fMRI.
