# Supplementary figures and images for: Temporoparietal brain structures support sentence processing across the adult lifespan
Source: Imaging Neurosci (Camb). 2025 Nov 13;3:IMAG.a.1009. doi: 10.1162/IMAG.a.1009 (PMC12616153; doi:10.1162/IMAG.a.1009)

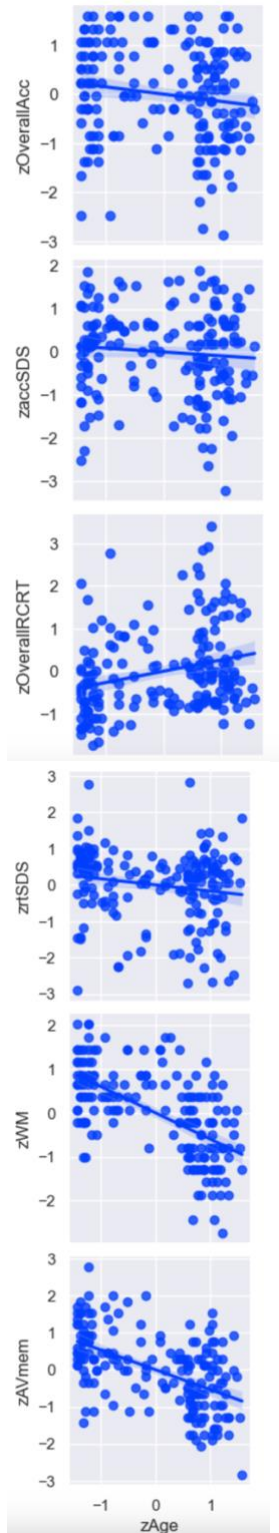

Supplementary Figure: Linear relationships between age and behavioral variables

Supplement: Supplementary Material [file IMAG.a.1009_supp.pdf]
